# Glacial meltwater determines the balance between autotrophic and heterotrophic processes in a Greenland fjord

**DOI:** 10.1073/pnas.2207024119

**Published:** 2022-12-19

**Authors:** Mikael K. Sejr, Annette Bruhn, Tage Dalsgaard, Thomas Juul-Pedersen, Colin A. Stedmon, Martin Blicher, Lorenz Meire, Kenneth D. Mankoff, Jakob Thyrring

**Affiliations:** ^a^Department of Ecoscience, Aarhus University, Aarhus DK-8000, Denmark; ^b^Arctic Research Centre, Aarhus University, Aarhus DK-8000, Denmark; ^c^Unisense A/S, Unisense A/S, Aarhus N DK-8200, Denmark; ^d^Greenland Climate Research Centre, Greenland Institute of Natural Resources, Nuuk 3900, Greenland; ^e^National Institute of Aquatic Resources, Technical University of Denmark, Kgs Lyngby 2800, Denmark; ^f^Department of Estuarine and Delta Systems, Royal Netherlands Institute for Sea Research, Yerseke 4400, The Netherlands; ^g^Department of Glaciology and Climate, Geological Survey of Denmark and Greenland, Copenhagen K 1350, Denmark

**Keywords:** Coastal ecology, Greenland, primary production, respiration, CO_2_

## Abstract

Global warming accelerates melting of glaciers and increases the supply of meltwater and associated inorganic particles, nutrients, and organic matter to adjacent coastal seas, but the ecosystem impact is poorly resolved and quantified. When meltwater is delivered by glacial rivers, the potential impact could be a reduction in light and nutrient availability for primary producers while supplying allochthonous carbon for heterotrophic processes, thereby tipping the net community metabolism toward heterotrophy. To test this hypothesis, we determined physical and biogeochemical parameters along a 110-km fjord transect in NE Greenland fjord, impacted by glacial meltwater from the Greenland Ice Sheet. The meltwater is delivered from glacier-fed river outlets in the inner parts of the fjord, creating a gradient in salinity and turbidity. The planktonic primary production was low, 20–45 mg C m^−2^ d^−1^, in the more turbid inner half of the fjord, increasing 10-fold to around 350 mg C m^−2^ d^−1^ in the shelf waters outside the fjord. Plankton community metabolism was measured at three stations, which displayed a transition from net heterotrophy in the inner fjord to net autotrophy in the coastal shelf waters. Respiration was significantly correlated to turbidity, with a 10-fold increase in the inner turbid part of the fjord. We estimated the changes in meltwater input and sea ice coverage in the area for the last 60 y. The long-term trend and the observed effects demonstrated the importance of freshwater runoff as a key driver of coastal ecosystem change in the Arctic with potential negative consequences for coastal productivity.

## Significance

As the Arctic continues to warm, increased precipitation and melting of glaciers result in more freshwater runoff to the coastal ocean. This intensifies the coupling between land and ocean with consequences for marine ecosystems along the expansive Arctic coastline. We show that in parts of a Greenland fjord most impacted by meltwater, the rates of bacterial degradation exceed that of new production by phytoplankton, resulting in a net heterotrophic ecosystem. With increasing distance from the Greenland Ice Sheet, we find a gradual transition to an autotrophic system in the outer fjord where production exceeds degradation. Our finding is an example of how meltwater influences key ecosystem processes and strongly suggests that increasing runoff to Greenland fjords has consequences for coastal productivity.

Increasing temperatures induce melting of the Arctic cryosphere including permafrost, glaciers, and sea ice. Combined with increased precipitation, it leads to increased fluxes of freshwater and associated terrigenous material, including organic matter and nutrients (1–3). The combination of warming and freshening has the potential to increase the degree of stratification in open and coastal waters and is hereby expected to impact the status of marine ecosystems. Freshening is especially relevant in the coastal waters around Greenland where the mass loss of the Greenland ice sheet has increased sixfold compared to the 1980s (4), and the downstream effects of high-latitude freshening have been documented (5). The ecosystem consequences of increasing input of freshwater to the Arctic seas in general and the Greenland fjords in particular are poorly quantified. Arctic freshening is a complex process impacting physical and biogeochemical properties and resulting in a broad range of interconnected impacts to biota, making the cumulative impacts on the ecosystems difficult to predict. In the open ocean, a key impact of freshening is increased stratification which may limit nutrient replenishment to the photic zone during postbloom conditions as has been observed in the Canada Basin (6). Freshening is also associated with a decrease in nutrient concentration in surface layers of the central Arctic Ocean (7), increased prokaryote production (8), and a shift in primary producers toward a dominance of picophytoplankton (9). On a more local scale, the freshwater entering Greenland fjords is known to create distinct physical conditions in the inner fjords resulting in biogeochemical gradients (10), inducing physiological stress in residing organisms, and changes in pelagic and benthic community composition (11, 12). As for coastal ecosystems in general, the impact of freshwater depends on local conditions such as catchment area and characteristics, bathymetry (sill depth in particular), and tidal mixing (13). However, a defining characteristic of fjords with marine-terminating glaciers is that part of freshwater supply can be delivered as subglacial discharge and enter the fjord at depth. This results in a freshwater plume rising toward the surface, potentially maintaining a flux of nutrients from bottom waters to the photic zone (14). This has been shown to be important for some productive fjord systems in Greenland (15). In contrast, fjords with land-terminating glaciers, where freshwater is delivered at the fjord surface by rivers, can be highly turbid and stratified, resulting in poor light and nutrient availability and as a consequence, less productivity (16). In addition, glacial meltwater contains bioavailable organic carbon and nutrients which have been speculated to be important for coastal carbon cycling in both Greenland and Alaskan waters (17–19). The Arctic region in general is characterized by terrestrial input of carbon from several large rivers supplying allochthonous carbon from land to coastal regions. Further release of large stores of carbon from the surrounding land may have the potential to alter the Arctic Ocean from a CO_2_ sink to a source in future (20). The net effect of Greenlandic fjords on regional carbon budgets is currently unknown. Along Greenlands extensive coastline, there is a large number of fjords with variable impacts of meltwater, glaciers, and tidal exchange. In addition to this, it is unclear how changing conditions are influencing the biogeochemistry and as a consequence, the biological productivity. Studies from Siberian coastal waters with extensive input of terrestrial carbon show that mineralization of terrigenous organic matter maintains a net heterotrophic system, releasing CO_2_ to the atmosphere (21). In general, fjords in West Greenland are productive with tidal mixing and marine-terminating glaciers, supporting vertical mixing of nutrients (15). Here, fjords are essentially autotrophic for most of the year and function as a sink for atmospheric CO_2_ (22, 23). For these systems, the concentrations of the bioavailable dissolved organic carbon (DOC) in the local rivers can be significantly lower than those reported for Alaskan glaciers (17) and also lower than concentrations generally found in fjord waters (24), suggesting the allochthonous input to be low. The situation could be different in East Greenland fjords, such as Young Sound. It receives 0.63–1.57 km^3^ of freshwater annually from melting sea ice, land-terminating glaciers, and seasonal precipitation in its catchment (25). During the ice-free period (mid-July to mid-October), the fjord is highly stratified and the primary production in the outer fjord is limited to 10 mg C m^−2^ y^−1^ (26). Sediment trap measurements indicate that the majority of particulate organic carbon (POC) reaching the sea floor may be of terrestrial origin (27). The short growth season, low nutrient concentrations, and weak vertical mixing combined with river input make this fjord a site where allochthonous carbon in general and carbon related to glacial meltwater in particular could be important. The aim of this study was to investigate how glacial and terrestrial meltwater may influence coastal pelagic carbon cycling and the potential for uptake of atmospheric CO_2_ using a unique suite of seasonal measurements along a 120-km transect extending through Young Sound and onto the shelf of the NE Greenland coast.

## Materials and Methods

### Study Site.

**Fig. 1.**
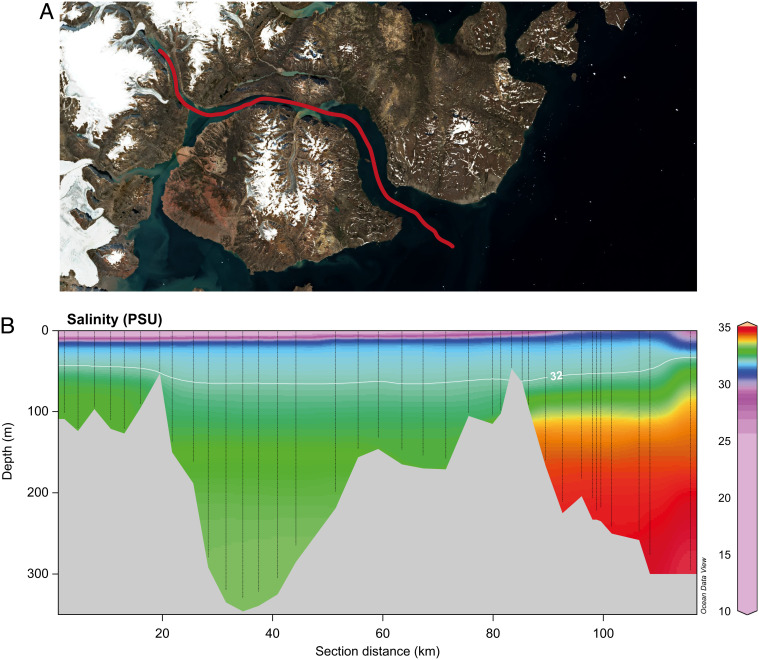
(*A*) Map of Young Sound with transect. Satellite photo from August 15, 2021. (*B*) Contour plot of salinity along the transect line.

Young Sound is a high Arctic sill fjord in NE Greenland (74°18′N, 20°18′W) (Fig. 1). The fjord can be divided into three sections: the deep and narrow inner fjord (Tyrolerfjord), the central fjord between Wollaston Foreland and Clavering Island, and the outer fjord connecting to the Greenland Sea. The outer sill depth is 45 m and maximum depth in the central basin is 325 m. The fjord system is typically covered by sea ice nine months a year (October to June). The freshwater inputs are derived from meltwater from the local glaciers and the Greenland ice sheet, runoff from precipitation in nonglaciated areas, as well as melted sea ice. Glacial meltwater has been estimated to contribute around 50–80% of the total runoff from the catchment (28). The total catchment area is 3,016 km^2^, and the freshwater input is estimated to be 0.630–1.570 km^3^, approximately shared by a ratio of 3:1:1 between the three subcatchment areas; the land-terminating glaciers in the bottom of Tyrolerfjord feeding the Tyroler river, the Zackenberg river, and the Lerbugt river (29).

### Sampling.

Sampling was carried out between July 27 and August 16, 2011, during the time of peak freshwater discharge. Physical, chemical, and biological data were collected at a total of 10 sampling stations distributed along a transect from Tyrolerfjord and 20 km out into the East Greenland shelf (Fig. 1). At an additional 27 stations, vertical profiles of temperature, salinity, chlorophyll fluorescence, photosynthetic available radiation (PAR), turbidity, and dissolved oxygen were obtained using a conductivity, temperature, depth (CTD) profiler (Sea Bird, SBE19+) with a light meter (PAR, Li-Cor 190SA quantum Q, Li-Cor). A 5-L Niskin bottle was used to collect water samples from depths of 5, 10, 20, 30, 40, and 80 m as well as from the deep chlorophyll maximum (DCM) when present. Samples for dissolved inorganic nutrients were taken from all depths, whereas phytoplankton composition, chlorophyll *a*, particulate and DOC, and ^14^C primary production were analyzed for the upper 40 m. Partial pressure of CO_2_ (pCO_2_) was measured in the surface water only (0.5 m).

An oceanographic mooring was deployed from August 2011 to August 2012 in the outer part of the fjord (Fig. 1). Three CTDs (Sea Bird SBE-37) were mounted at the mooring at depths of 17, 35, and 65 m, programmed for logging salinity, pressure, and temperature every 20 min. The CTD at 17 m was also equipped with sensors for PAR and chlorophyll fluorescence. An autonomous rotating sediment trap (Technicap) was mounted on the mooring at 65 m depth.

### Nutrients, Carbon Pools, and Chlorophyll.

The concentrations of NO_3_^−^ + NO_2_^−^ were determined as NO on a NOx analyzer (Model 42C, Thermo Environmental Instruments) after reduction to NO in hot vanadium chloride (30). Phosphate and silicate were determined by standard colorimetric methods and analyzed automatically on a robotic sample processor coupled to a spectrophotometer (Gilson 222 XL & Shimadzu 1600 PC). Detection limits for NO_3_^−^ + NO_2_^−^, phosphate, and silicate were 0.15, 0.20, and 0.25 μmol L^−1^, respectively.

The partial pCO_2_ was estimated by passing water from a Niskin bottle through Tygon tubing into a membrane equilibrator (Mini Module, Liqui-cel). Air was circulated in a closed system between the equilibrator and an infrared CO_2_ analyzer (Environmental Gas Monitor EGM-4, PP Systems) until a stable reading was obtained.

Size-fractionated chlorophyll *a* concentrations were determined by filtration (max 0.3 bar) of known volumes of seawater onto four different filters types: GF/C and GF/F (Whatman, Sigma-Aldrich, nominal pore size 0.7 and 1.2 μm, respectively), as well as filters with a pore size of 10 and 50 µm (Aquanet, Haukrogh, Denmark). Triplicate water samples from each sampling depth were filtered onto each type of filter. Pigments were extracted in 96% ethanol (6 to 24 h in darkness at 4°C), and fluorescence of the extracts was measured using a Turner ® Trilogy Laboratory fluorometer (Turner Designs) before and after addition of 3 drops of 0.1 N HCl. Concentrations of chlorophyll *a* were calculated according to a standard curve based on a pure chlorophyll *a* standard (DHI LAB products, Denmark). The taxonomic composition of protist communities was determined from water samples fixed in acidic Lugol’s solution (final concentration of 2%). The samples were kept cool and dark until analyses (max. of 6 mo). Depending on the cell concentrations, 50 to 100 mL subsamples were allowed to settle for 24 h in sedimentation chambers. All (or a minimum of 300) cells were counted using an inverted microscope.

### Community Production and Respiration.

Production and consumption of O_2_ was determined at three stations by light and dark incubations in 120 mL Winkler bottles in order to determine net community production (NCP) and community respiration (CR). Gross primary production was estimated as the sum of NCP and CR. A 10-L polyethylene container was filled with water from each incubation depth (1, 10, 20, 40, and 80 m) collected using a 5-L Niskin bottle. The container was gently rotated to homogenize the water before it was siphoned into 24 Winkler bottles through a piece of Tygon tubing, allowing the water to overflow for two volume changes. During this process, the water was filtered through a 200-µm filter to remove any metazoan plankton. The volume of the bottles was ca. 120 mL, but the exact volume of each was used in the calculations. Eight of these received Winkler reagents I and II (720 µL each) immediately for determination of the initial O_2_ concentrations, eight others were placed inside two closed gray PVC tubes for incubation in darkness, and the last eight were placed inside two Plexiglass tubes for incubation in light (at 80 m, only the dark incubation was performed). The tubes with bottles were deployed at sampling station and depth for 24 h on a mooring. The incubated bottles received Winkler reagents I and II immediately after being retrieved. The O_2_ concentration was analyzed with the photometric Winkler technique (31) as modified by (32). Absorbance was read at 466 nm on a Shimadzu 1240 spectrophotometer recording four readings for each bottle. The results are presented with propagated standard deviations.

### Primary Production.

Primary production was estimated by the ^14^C incubation technique (33). Samples from 5, 10, 20, 30, and 40 m depth were incubated in situ in 110 mL glass bottles (2 light and 1 dark bottles at each depth) for 2 h around noon. After incubation, the samples were transported in darkness to the lab and filtered on GF/F filters. 100 μL 1M HCl was added to each filter to remove excess ^14^C. Filters were fumed for a minimum of 12 h. The samples were analyzed on a scintillation analyzer (PerkinElmer) after addition of scintillation fluid (TriCarb 2800 TR). Production values were corrected for dark uptake. Samples for total inorganic carbon used for the calculation of primary production were collected in 12 mL glass vials from the same depths as primary productivity. The samples were poisoned immediately and analyzed coulometrically. Daily production values were calculated by multiplying productivity during the 2 h incubations with the ratio between incoming PAR during the incubation and average daily PAR (data obtained from the ClimateBasis monitoring program; https://data.g-e-m.dk).

### Long-Term Changes in Sea Ice and Runoff.

Sea ice cover in outer Young Sound is based on on-site observations by the Royal Danish Navy at Daneborg as reported by Rysgaard & Glud (2003) and has since 2003 been updated by the Greenland Environmental Monitoring (GEM) Program based on daily images by an autonomous camera system. Runoff was estimated by extracting all modeled runoff that drains through outlets located between 74 and 75°N from the Regional Atmospheric Climate Model (RACMO). (34). The RACMO provides both ice and land runoff. The ice runoff is dominated by melted ice, but also includes treatment of rainfall, retention, and refreezing (35). The RCM is validated against observations elsewhere (34) and has an uncertainty of approximately a factor of two when averaged annually in time and at basin scale spatially. As the work done here averages annually in time and by 1 degree spatially, uncertainty should be somewhat less than a factor of two.

### Calculations, Definitions, and Statistical Analysis.

The photic depth (Z_eu_) was defined as the depth of 0.2% surface irradiance, based on the PAR attenuation (K_d_), i.e., Z_eu_ = ln(100/0.2)/K_d_ = 6.215/K_d_. The stratification index of the upper water column was estimated as the density difference between 1 and 80 m depth. The nitracline was defined as the shallowest depth where the measured concentration of nitrate plus nitrite was >0.1 μM. A correlation matrix based on data collected at the 10 sampling stations was created using the reshape2 (36) and ggplot2 (37) packages in the R program (38). A generalized linear model (GLM) was used to model correlations between environmental data from the summer sampling campaign 2011 and primary production (Table 1). The full dataset included NO_3_^−^, PO_4_, Si(OH)_4_, temperature, and PAR at the depth of the photic zone. Prior to analysis, a data exploration process was carried out (39). Scatterplots were used to identify potential outliers, and relationships between covariates were assessed using Pearson’s correlation coefficients. This revealed collinearity between Si(OH)_4_ and NO_3_^−^ (*r* = 0.71) and PO_4_ (*r* = 0.70), a pattern supported by the variance inflation factor value of Si(OH)_4_ (VIF = 4.19; threshold <3; (40). Based on the correlation coefficients and VIF, Si(OH)_4_ was excluded from the model to eliminate correlation between covariates. The data were analyzed using a GLM with a Gaussian distribution on log-transformed primary production data to ensure acceptable residual patterns. The models were reduced to final best-fit models using Akaike Information Criterion (AIC) with ΔAIC < 2. The final model contained temperature and PAR as explanatory variables, and the model was validated by inspecting the standardized residual patterns plotted against fitted values.

**Table 1.** Environmental variables of the water column in Young Sound during the summer sampling campaign 2011

Sampling station	Δσ_t_	Z_eu_ (m)	T_eu_ (°C)	S_eu_	Z_nit_ (m)	Z_DCM_	NO_3_+NO_2_ (µM)	Si(OH)_4_ (µM)	PO_4_ (µM)	N:P	N:Si
TYRO-1	24.85	4.1	5.43	20.8	10	1	0.39	9.37	1.28	0.30	0.04
TYRO-5	24.75	7.9	3.46	29.7	20	10	0.00	1.02	0.50	0.01	0.00
TYRO-10	21.02	35.8	0.41	28.6	30	20	0.00	3.45	0.47	0.00	0.00
TYRO-13	20.34	23.7	1.46	29.8	25	25	0.49	1.31	0.59	0.82	0.37
YS 3.18	17.73	33.4	0.67	30.9	30	30	0.49	1.48	0.58	0.84	0.33
YS 3.12	8.17	34.7	−0.04	31.1	30	30	1.49	0.43	0.52	2.85	3.50
Main st.	5.45	41.6	−0.65	31.1	30	20	0.00	1.16	0.47	0.00	0.00
YS 3.03	5.36	43.5	−0.78	31.4	0	30	0.67	0.58	0.52	1.28	1.15
GH05	3.86	43.6	−1.21	31.6	10	26	0.89	0.73	0.54	1.63	1.21
GH08	3.17	47.8	−1.28	29.0	20	10	0.00	2.38	0.60	0.00	0.00

Δσ_t_: stratification index; Z_eu_: depth of the euphotic zone (0.2% of PAR); T_eu_: water temperature averaged over the photic zone; S_eu_: salinity averaged over the photic zone; Z_nit_: depth of the nitracline; Z_DCM_: depth of the deep chlorophyll maximum; NO_3_+NO_2_ nitrate+nitrite concentration at Z_DCM_; Si(OH)_4_: silicic acid concentration at Z_DCM_; PO_4_: phosphate concentration at Z_DCM_; N:P: molar ratio between nitrate+nitrite and phosphate at Z_DCM_; N:Si: molar ratio between nitrate+nitrite and Si at Z_DCM_.

## Results

### Water Column Structure and Production.

**Fig. 2.**
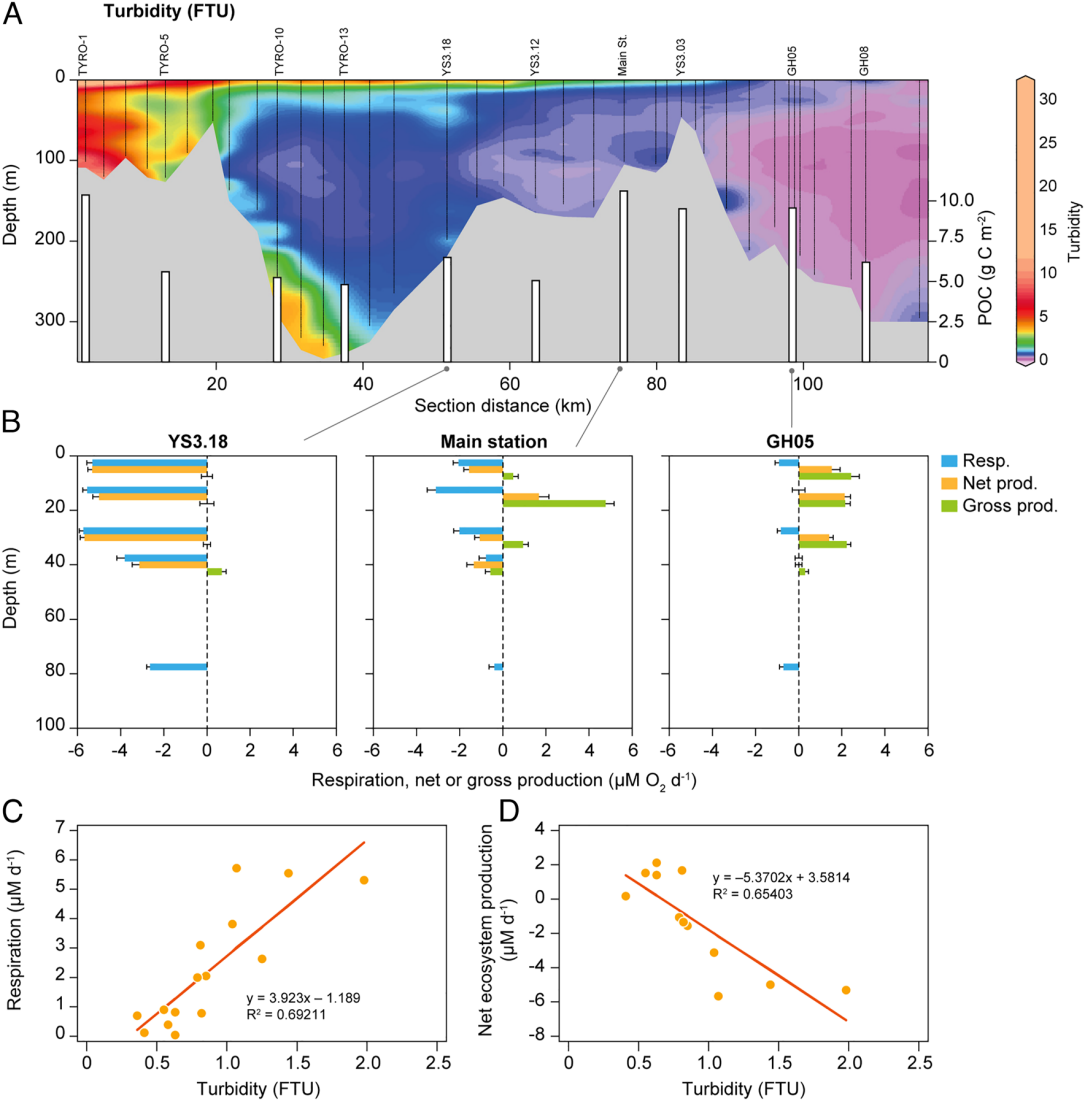
(*A*) Turbidity (contour plot) and integrated POC in the water column (bars). (*B*) Pelagic respiration and net and gross production at three stations in Young Sound. (*C*) Relationship between turbidity and respiration. (*D*) Relationship between turbidity and net ecosystem production.

During the sampling campaign, a distinct surface freshwater lens was observed throughout the fjord system, strongest in the inner part of the fjord (Fig. 1*B*). The degree of stratification decreased from the inner fjord toward the shelf waters (the stratification index decreased from ~25 to 3). In the photic zone, average salinity increased from 20.8 to 31.6 and temperature decreased from 5.43 to −1.28°C, along the transect toward the shelf (Table 1). The turbidity was highest in the inner part of the system which is influenced by glacial meltwater supplied by the Tyroler River. This resulted in high turbidity throughout the water column approximately 20 km downstream of the river mouth. In the central fjord, turbidity was confined to the surface layer (Fig. 2*A*). The amount of POC in the fjord showed two peaks (Fig. 2*A*); at the innermost part of the fjord where input of glacial meltwater was highest and near the outer sill related to a peak in chlorophyll *a* (see below), indicating that both allochthonous (related to the meltwater) and autochthonous sources (local production) were important. Along the gradient of glacial meltwater influence, the pelagic community metabolism changed significantly. At approximately 50 km from the head of the fjord (station Young Sound-3.18), the water column was net heterotrophic, with a maximum respiration of 4–6 μM O_2_ d^−1^ in the upper 40 m (Fig. 2*B*). Gross production was below detection levels except at 40 m. Moving ca. 20 km toward the sea, at the Main station (Fig. 2*B*), respiration rates were reduced to 1–3 μM O_2_ d^−1^, while gross production increased throughout the water column, leading to a net production of 5 μM O_2_ d^−1^ at 20 m depth. At the marine end of the transect (station GH05) in the Greenland Sea (Fig. 2*B*), pelagic respiration was below 1 μM O_2_ d^−1^ at all depths with net production down to 30 m and thus an autotrophic surface layer. A significant linear relationship was found between turbidity and respiration rates and net ecosystem metabolism across depths and stations (Fig. 2 *C* and *D*).

**Fig. 3.**
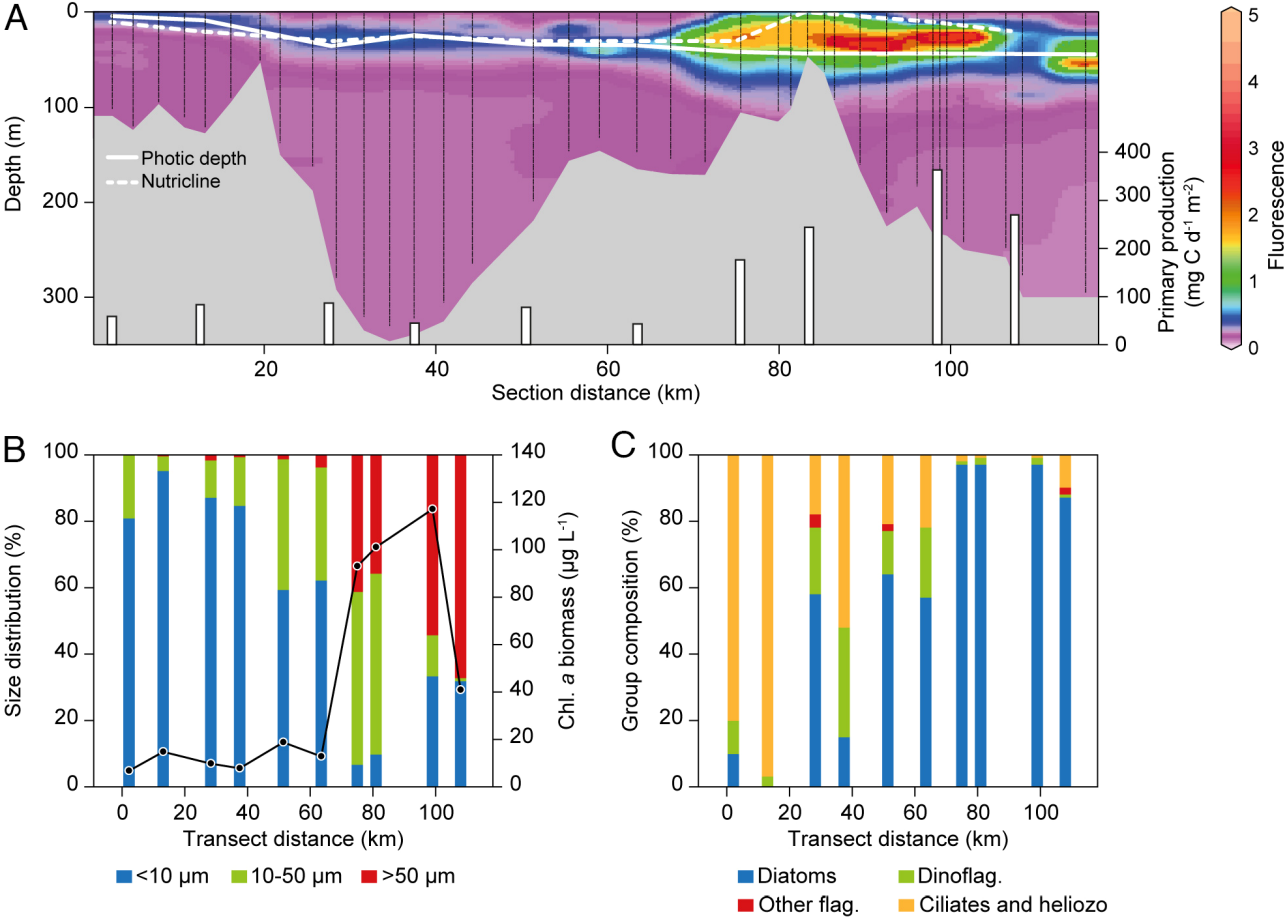
(*A*) Fluorescence (contour plot), primary production, ^14^C (bars) primary production, photic zone (solid white line), nitracline (dashed white line). (*B*) Size distribution of phytoplankton along the fjord transect and integrated Chl *a* biomass (solid black line). (*C*) The composition of the phytoplankton community.

The chlorophyll *a* (Chl *a*) fluorescence showed a gradually deepening maximum going from the head to the mouth of the fjord (Fig. 3*A*). The highest fluorescence was found near the shallow sill and extending into coastal shelf waters with maximum values at 10 to 50 m depth over a distance of 30 km. The pattern likely reflects the deepening photic zone and changes in the depth of the nitracline (Fig. 3*A*). The integrated Chl *a* increased from 7 mg Chl *a* m^−2^ at the inner station (TYRO_01) to a maximum of 118 mg Chl *a* m^−2^ at station GH05 (Table 2 and Fig. 3*B*). The size distribution of Chl *a* also changed along the fjord with a strong dominance of cells smaller than 10 μm in the inner 40 km of the fjord to a dominance of large cells (>50 μm) from the mouth of the fjord and into the Greenland Sea (Fig. 3*B*). This transition in size is also reflected in the taxonomic composition, where ciliates and heliozoans dominated the inner fjord, but were gradually replaced by diatoms toward the sea (Fig. 3*C*). In the inner fjord, the vertical extent of the photic zone increased from 4 to 24 m (Fig. 3*A*). For all stations in this part of the fjord, except for TYRO-10, the nitracline (>0.1 µM NO_3_+NO_2_) was generally found 5–10 m below the photic zone. In the central and outer fjord, the photic zone gradually deepened to 48 m depth on the shelf and the nitricline also deepened, however remained in the photic zone (Table 1). Phosphate concentrations were elevated in the surface waters of the inner fjord (1.3 µM) due to the freshwater inputs. In the central and outer fjord, phosphate was more homogeneously distributed vertically at all stations, with concentrations ranging between 0.4 and 0.6 µM. Silicate concentrations were on the whole highest throughout the water column in the inner fjord and depleted in the surface waters of the central fjord and, as for nitrate, they increased in the bottom waters going toward the shelf. At the depth of the DCM, molar ratios of N:P and N:Si of 0–2.85 and 0–3.5, respectively, indicated that N generally was the primary limiting nutrient here, but also that Si could be limiting diatom production (YS 3.12, YS 3.03, and GH05).

**Table 2.** Biological variables at the 10 sampling stations in Young Sound in summer 2011

	PP	Integrated biomass				Productivity	Taxonomic composition (% of total abundance at DCM)			
Sampling station	(mg C m^−2^ d^−1^)	B_T_ (mg chl a m^−2^)	B_S_ (%)	B_L_ (%)	B_VL_ (%)	(mg C m^−2^ d^−2^ mg chl a^−1^)	Dia	Dino	Other flag	Cil+Hel
TYRO-1	30	7.0	80.8	19.1	0.1	4.2	10	10	0	80
TYRO-5	42	15.2	95.1	4.4	0.6	2.7	0	3.2	0	96.8
TYRO-10	43	9.8	87.0	11.2	1.8	4.4	57.7	19.2	3.8	19.2
TYRO-13	29	8.3	84.6	14.6	0.8	2.7	14.5	32.7	0.0	52.7
YS 3.18	39	19.1	59.3	39.3	1.4	2.1	64.2	13.1	2.2	20.4
YS 3.12	22	12.7	62.2	34.0	3.8	1.7	57.3	20.9	0.0	21.8
Main st.	153	93.0	6.8	52.0	41.2	1.6	97.5	1.3	0.0	1.3
YS 3.03	244	101.9	9.9	54.4	35.8	2.4	97.2	1.7	0.0	1.1
GH05	368	117.8	33.4	12.4	54.3	3.1	96.6	1.9	0.8	0.8
GH08	273	41.6	31.9	0.9	67.2	6.6	87.1	1.0	1.4	10.5

PP: primary production integrated over the water column; B_T_: total biomass integrated over the water column; B_S_: percentage of B_T_ of small cells (1.3–10 µm); B_L_: percentage of B_T_ of large cells (10–50 µm); B_VL_: percentage of B_T_ of very large cells (>50 µm); Productivity: PP per unit of chl *a* integrated over the water column. Taxonomic composition of the community at the DCM. Diatoms (Dia), dinoflagellates (Dino), other flagellates (Other flag), and Ciliates + Heliozoans (Cil+Hel).

**Fig. 4.**
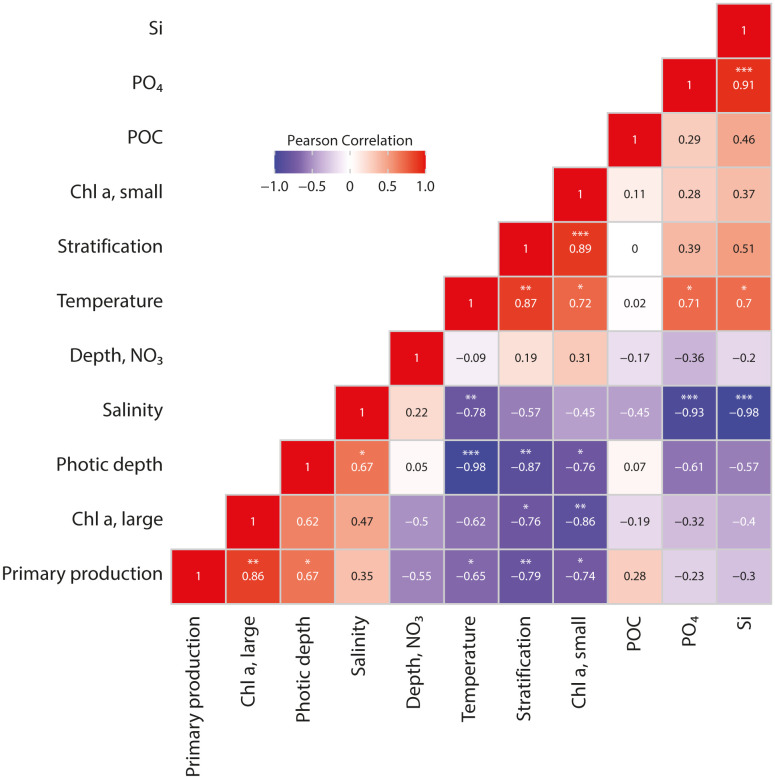
Pearson’s correlations among integrated primary production and physical, chemical, and biological variables at 10 stations in Young Sound. Positive correlations are shown in blue; negative correlations are shown in red.

The integrated primary production was generally low (less than 50 mg C m^−2^ d^−1^) in the inner 65 km of the fjord, but increased over the sill and in shelf waters to a maximum of 368 mg C m^−2^ d^−1^ at station GH05 (Table 2 and Fig. 3*A*). The GLM analysis of the depth-specific primary production showed a significant effect of light (quantified as %PAR left compared to surface) and a marginal insignificant relationship with temperature (Table 3). The integrated primary production showed a significant positive correlation to the integrated Chl *a* biomass and depth of the photic zone and a negative correlation to temperature, the stratification index, and the proportion of small phytoplankton cells (Fig. 4).

**Table 3.** Generalized linear model results

GLM model (Gaussian distribution)				
	Estimate	SE	*t*-value	*P-*value
(Intercept)	−1.24	0.39	−3.17	**0.003**
PAR	0.08	0.02	3.92	**<0.001**
Temperature	−0.55	0.27	−2.01	0.05

Estimated parameters, SE, *t*-values, and *P*-values (significance level *P* < 0.05 in bold) from the final model of the relationship between primary production (C/m3/day), photosynthetic available radiation (PAR), and temperature (°C).

### Seasonal Pattern in Water Column Structure, Light, and Vertical Flux.

**Fig. 5.**
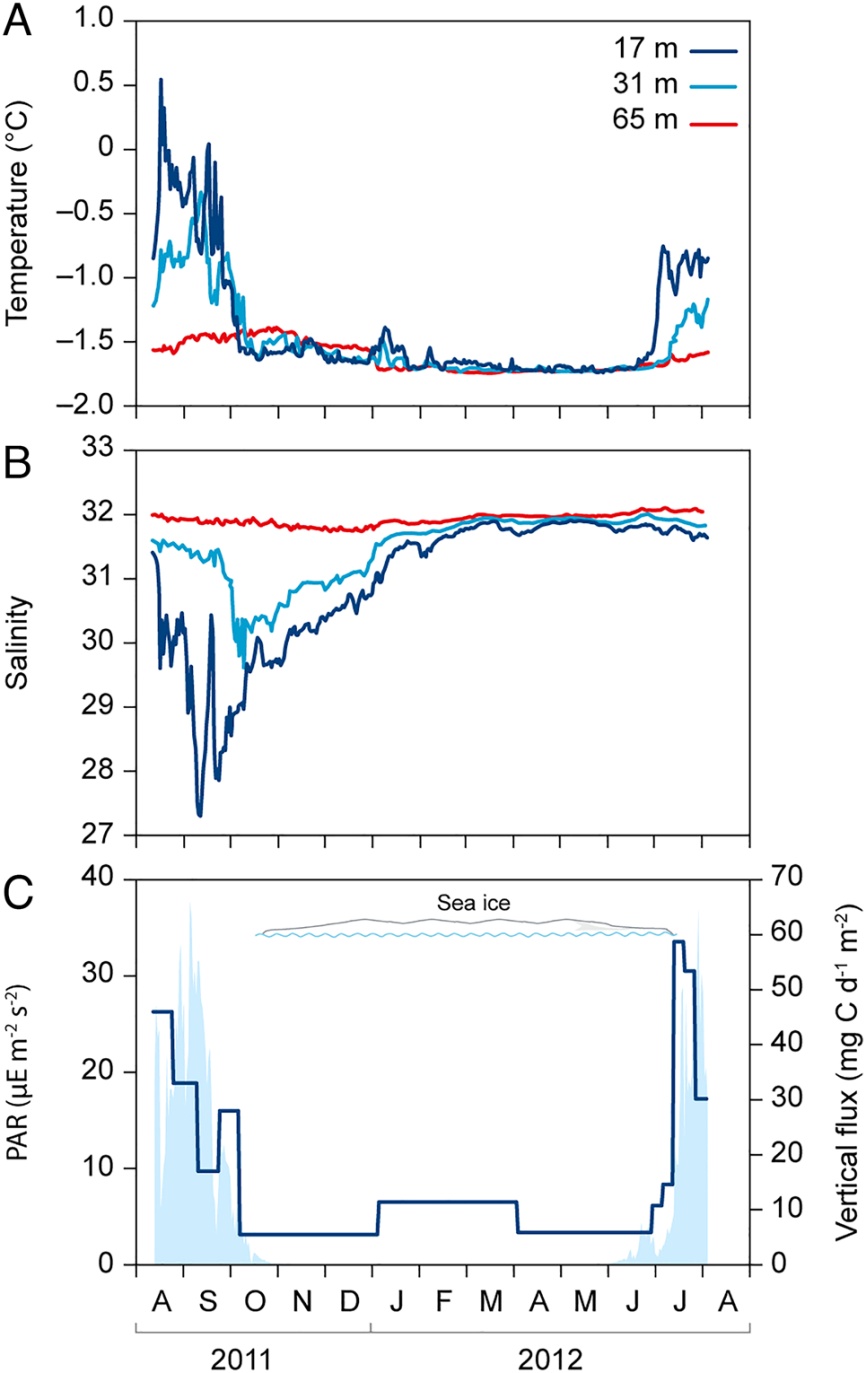
Data from seasonal mooring measured from August 2011 to August 2012. (*A*) Temperature and salinity (*B*) at three different depths. (*C*) PAR data from 17 m depth (light blue shade) and vertical flux of carbon (dark blue line).

The mooring in outer Young Sound provided a basic description of the conditions in the fjord outside the sampling campaign in August 2011 (Fig. 5). The rapid changes in water column structure during mid-August 2011, just after the sampling campaign, reflected the summer/autumn downward mixing of the shallow surface layer of low-saline warmer water. This downward heat flux resulted in maximum annual temperature at 65 m to occur around November 1. However, the decrease in salinity at 65 m depth persisted until January. In spring 2012, heating at 17 m could be observed from late June, when this part of the fjord was still ice covered, indicating advection of warmer water from outside the fjord which has areas of open water during most of the year. The vertical flux of POC was highest during July and August (30–59 mg), peaked just prior to ice breakup in June 2012. The C:N ratio of the sedimented organic material decreased from 13 to 10 over the autumn, but was at the lowest in the early summer, in a short period decreasing below 9 (Table 4). During autumn and winter, the trapped carbon had a δ^13^C signature of approximately −24, whereas the C sedimented during the early summer has a significantly lower ^13^C signature of between −27 and −26 (Table 4).

**Table 4.** Vertical flux of total particulate matter and total particulate carbon, and C:N ratio and δ13C signature of the sedimented material during the deployment of an autonomous rotating sediment trap (Technicap; 12 bottle system) at 65 m depth.

Dates (start - end)	Duration (days)	Total particulate matter (g m^−2^ d^−1^)	Total particulate carbon (mg C m^−2^ d^−1^)	C:N ratio (mol:mol)	δ^13^ C (‰)
12/08/2011 – 26/08/2011	14	2.03	46.64	12.24	−24.12
26/08/2011 – 09/09/2011	14	1.09	33.33	10.53	−24.09
09/09/2011 – 23/09/2011	14	0.54	17.13	10.82	−24.02
23/09/2011 – 07/10/2011	14	1.06	28.94	9.98	−23.34
07/10/2011 – 05/01/2012	90	0.21	5.49	10.22	−23.72
05/01/2012 – 04/04/2012	90	0.61	11.41	11.52	−23.58
04/04/2012 – 30/06/2012	87	0.31	5.90	13.07	−23.94
30/06/2012 – 07/07/2012	7	0.15	10.77	11.35	−25.63
07/07/2012 – 14/07/2012	7	0.19	14.60	8.78	−25.45
14/07/2012 – 21/07/2012	7	1.22	58.66	13.11	−26.82
21/07/2012 – 28/07/2012	7	2.02	53.42	12.39	−26.56
28/07/2012 – 04/08/2012	7	0.84	30.16	11.79	−26.71
12/08/2011 – 04/08/2012	359	199.33^*^	4984.53^*^	11.32^†^	−24.83

^*^Total sinking flux during the deployment period (359 d).

^†^Average value during the deployment period (359 d).

### Surface Water pCO_2_.

**Fig. 6.**
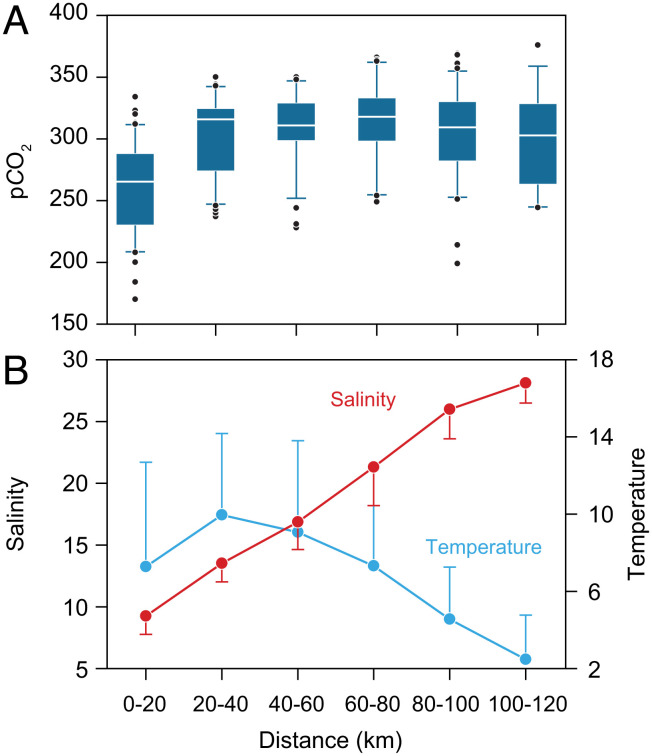
(*A*) Average (2006–2013) summer surface (0–1 m) pCO_2_ in the Young Sound, NE Greenland from the inner fjord, near the Greenland Ice Sheet (0–20 km) to the Greenland Sea (100–120 km). (*B*) Average summer salinity and temperature of the surface waters (1 m depth) of Young Sound (average ± SD).

The partial pCO_2_ in the surface waters at all sampling sites was below atmospheric saturation during the sampling campaign. Using data from the monitoring program allows us to calculate the average surface conditions in the fjord during late July and early August from 2008 to 2013 (Fig. 6*A*). Every year, a similar pattern was observed with low salinities in the inner fjord and a gradual increase toward the mouth of the fjord (Fig. 6*B*). Surface temperature reached 10°C in the inner part of the fjord, but decreased toward the sea (Fig. 6*B*).

### Long-Term Changes in Sea Ice and Runoff.

**Fig. 7.**
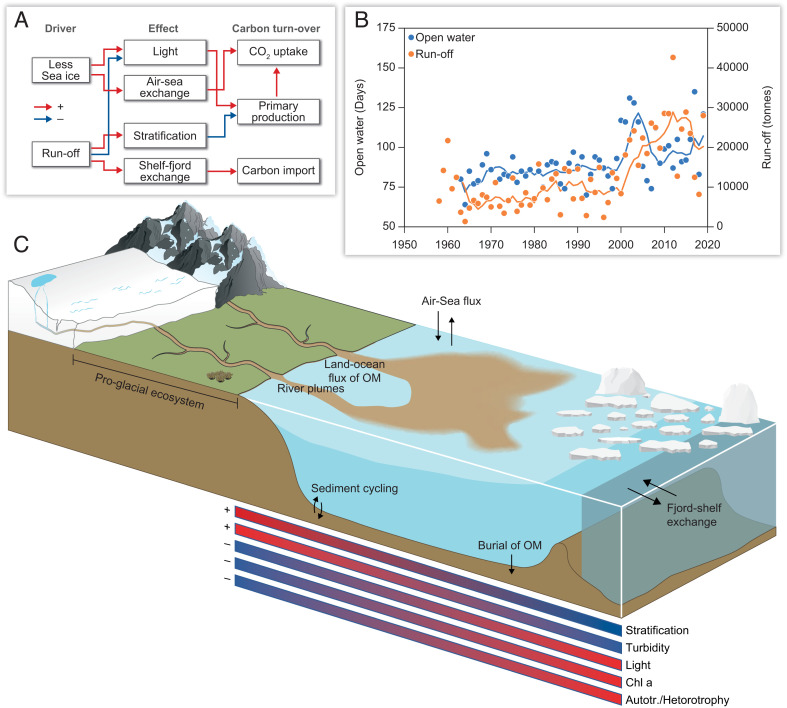
(*A*) Conceptual diagram of the main effects of changes in runoff and sea ice cover on carbon cycling in Young Sound during summer. (*B*) Changes in days with open water (no sea ice) and runoff from land since 1960 including the 5-y running mean for each. (*C*) A schematic figure showing spatial gradients observed in Young Sound in summer.

The number of days with no sea ice/open water per year shows a slow but constant increase for about 40 y until 2000, after which the interannual variability increases as does the rate of increase based on the 5-y running mean (Fig. 7). The regional runoff shows a similar trend, with an apparent increase in both variability and rate of change around 2000. The relative change in runoff is higher than the change in days with open water, with the change after 2000 exceeding a doubling compared to the beginning of the time series.

## Discussion

### Runoff and Pelagic Ecosystem Metabolism.

The present data collected during post spring bloom conditions in late July and early August show a strong impact of glacial river runoff on physical and biogeochemical conditions in the fjord. At the inner station with the lowest surface salinity, pelagic respiration is stimulated while gross production is very low. In contrast, respiration is lower outside the fjord and gross production is greater, indicating that along the fjord there is a transition from net heterotrophic in the inner part to net autotrophic in the outer part. Previous studies in Greenland and Alaska have shown glacial meltwater to deliver bioavailable DOC and argued that glacial-derived carbon could be an important source of allochthonous carbon that could influence carbon cycling in coastal ecosystems (17, 41, 42). Organic carbon delivered by glacial meltwater could contribute to the increased respiration observed in the inner fjord. However, without trace data such as ^14^C date of the carbon in the fjord, it cannot be quantified if the carbon originates from land or glaciers. We found an increase in water column content of POC in the region most influenced by freshwater, where primary production was very low, suggesting that rivers could contribute both particulate and dissolved carbon. However, when glacial particles are exposed to marine waters, they aggregate and form larger particles, resulting in complex adsorption and desorption of organic and inorganic molecules (43). In a highly turbid fjord in West Greenland with low Chl *a* concentrations, the organic fraction of particles (loss from ignition) was about 2% near the river but increased to 16–55% some 80 km downstream (44) and indicates adsorption of DOC to glacial particles. A similar process might occur in our study and explain the positive relationship between respiration and turbidity.

Previous studies in Young Sound estimated that in the outer fjord (approximately corresponding to 60 to 80 km in Fig. 1*B*), the input of total organic carbon from rivers amounted to 900 t C y^−1^ compared to an annual primary production of 1119 t C y^−1^ (45), which is equivalent to 12 g C m^−2^ y^−1^. A study by Lawson et al. (2014) estimated that the total carbon transport from the Greenland ice sheet with meltwater was in the range of 0.13 – 0.17 Tg C y^−1^ for DOC and 0.36 – 1.52 Tg C y^−1^ for POC. Using an average export of 1 Tg C, this results in an average loading of 30 g C m^−2^ y^−1^ if equally distributed in Greenland fjords using a total area of 32.900 km^2^ for Greenland fjords (46). These rough estimates suggest that the carbon loading from terrestrial/glacial sources in Young Sound may be slightly lower than that in other fjord systems impacted by meltwater from the Greenland Ice Sheet. Consequently, net heterotrophy is potentially a recurrent feature in NE Greenland fjords with high turbidity and low productivity during summer. The importance of terrestrial/glacial carbon in Young Sound was also shown in a seasonal (July to October) study of bacterial production, where the carbon demand of the bacterioplankton exceeded the pelagic primary production (19). However, there is an important distinction between the POC and DOC dynamics in the fjord; the freshwater from land is very low in DOC, and the coastal ocean is therefore a more important source for DOC (41). Alternative factors than carbon supply could also stimulate the pelagic respiration in the inner fjord. We found a significant relationship between respiration and turbidity but not between respiration and salinity; this couples the respiration to particles rather than the freshwater itself. A small fraction of the particles are organic, but small inorganic particles (glacial flour) are known to dominate in water draining glacial watersheds. These particles provide a large surface area for bacteria and the particles could also be partially primed with bacteria when entering the ocean, thus providing hot spots of bacterial abundance able to consume available carbon (47, 48). There are a limited amount of respiration data available from the Arctic coastal sites, but it is notable that respiration in inner Young Sound is higher than rates from a sub-Arctic Greenland fjord where gross productivity was 5 times higher than in Young Sound (22). It should also be noted that respiration measured in darkness, as in this study, has been shown to underestimate total light respiration (49).

In addition to delivering carbon to the fjord, meltwater with its content of inorganic particles also strongly affects availability of light, nutrients, and stratification. In Young Sound where surrounding glaciers all terminate on land and all the meltwater is delivered to the surface via rivers, the conditions for primary production are poor; the light penetration is limited; and the strong stratification limits replenishments of especially nitrate to the photic zone and results in low phytoplankton biomass (chlorophyll *a*), very low primary production, and a dominance of small primary producers (9). The low productivity in Young Sound has previously been attributed to the prolonged period of ice cover (45). Here, we show that meltwater from land strongly affects physical conditions in the fjord during ice-free summer with impacts on biogeochemical process rates and the composition of pelagic primary producers. This corresponds to a seasonal study, showing that meltwater and the associated turbidity limit light availability for primary production through most of August (16). The shallow photic zone, combined with strong stratification, weak vertical mixing, generally low nitrate concentrations, and extended ice coverage are thus all factors that contribute to the low autotrophic activity in the fjord.

Large-scale differences in coastal productivity in Greenland have previously been attributed to differences in sea ice cover (12, 50). But, a recent comparison between fjord systems with marine- and land-terminating glaciers has demonstrated the fundamental difference that glacial meltwater has on productivity (15). In fjords, such as Young Sound, land-terminating glaciers may restrict pelagic primary production by limiting nutrient supply and light penetration. In contrast, fjords with marine-terminating glaciers, a part of the meltwater, drain from beneath the glacial terminus, leading to upwelling in front of the glaciers. This upwelling creates hot spots of turbulence (51) and results in nutrient replenishment in the surface water, supporting high primary production and fishery near the glaciers (15). In Young Sound, we find maximum production in the outer region where the surface freshwater layer has partially disappeared and where a shallow sill with a maximum depth of 45 m contributes to vertical mixing (52). Sills are a characteristic feature in most Greenland fjords which combined with glaciers play an import role in shaping vertical mixing and connectivity to shelf waters.

### Seasonal Changes.

The carbon cycling described in this study reflects the period of Arctic summer, where influence of freshwater is at its maximum. Rivers into Young Sound typically start running in late-June and freeze up again in September (28). From the seasonal mooring, we see that stratification, as indicated by differences in salinity between the three depths, reaches a maximum in September as the surface water is gradually mixed down to 17 m depth. In October, a significant drop in salinity is seen at 31 m, indicating mixing related to autumn storms before the sea ice cover has formed. The seasonal variation in the vertical flux of carbon shows the combined effect of sea ice and runoff. The peak is seen just prior to ice breakup where meltwater ponds cover the sea ice, which increases the amount of light coming through the ice and fueling the spring bloom. The vertical flux of carbon during this period (June 30 to July 28) only represents 19% of the annual flux. During August and September, stratified conditions characterize the fjord, and nitrate concentrations are very low in most of the photic zone. The vertical particle flux is stable through this period; although average daily fluxes are only half of what was observed during the spring bloom, the vertical flux of carbon during August and September (July 28 to September 23) makes up 31% of the annual flux. The relatively high fluxes during autumn cannot be related to runoff, which cease in September and are likely driven by a combination of resuspension from storms and new production related to mixing. In Young Sound, local sea ice cover in the outer part of the fjord has been quantified since 1954. Although variability has generally increased in the last 10–15 y, the reduction in ice cover is relatively moderate with sea ice breaking up earlier and forming later in autumn (53). The later formation of sea ice in the Arctic in general has been argued to result in increased mixing from autumn storm, which could drive nutrient transport into surface waters and possibly sustain “autumn” blooms. Based on mooring data in Young Sound, the most pronounced change in sea ice conditions has been related to the timing of the ice to form in October where light availability is low, and based on the vertical flux of carbon, there is little evidence of increased productivity in autumn. Future changes in productivity in this fjord will thus also be determined by the quantity and timing of meltwater input and its influence and light and nutrient availability.

### Fjord Capacity for CO_2_ Uptake.

Greenland fjords, including Young Sound, have been shown to be consistently undersaturated in CO_2_ during summer and in some cases year-round (10, 23, 54). The 6 y of surface data from Young Sound show that the condition encountered during the 2011 field campaign represents typical conditions and that the observed gradient in surface salinity and temperature is a recurring feature. Mixing freshwater into seawater will in itself lead to undersaturation and has been estimated to drive 28% of the CO_2_ uptake in a SW Greenland fjord, with biological production being the dominant process (10). The much lower phytoplankton biomass and productivity in Young Sound combined with high meltwater input from rivers suggest that this physical mixing process combined with net heterotrophic biological input is important. Proglacial lakes and rivers have been shown to be undersaturated in CO_2_ due to chemical weathering, which can further contribute the undersaturation in glacial fjords (55).

The isolated surface lens with high amount of suspended particles means that sunlight is absorbed effectively, resulting in surface temperatures exceeding 14°C in summer. This leads to a reduction of the undersaturation in CO_2_ resulting in the nonlinear change observed along the salinity gradient in the fjord. The presence of undersaturated surface water despite the observation of net heterotrophy and thus biological production of CO_2_ combined with increasing pCO_2_ trend underlines the importance of physical factors influencing potential uptake of atmospheric CO_2_ in this coastal system. In contrast, other Arctic coastal systems such as the Laptev Sea receive a much larger input of allochthonous carbon from land, resulting in heterotrophy and outgassing of CO_2_ (56). The annual uptake of atmospheric CO_2_ has been estimated to be 32 g C m^−2^ y^−1^ in Young Sound (54) which when compared to an estimated annual primary production of 10 g C m^−2^ y^−1^ indicates that biologically driven CO_2_ dynamics is not the main control of the fjords’ capacity to take up atmospheric carbon. Although net heterotrophy in Young Sound and potentially in other Greenland fjords influenced by meltwater river is likely to persist during summer, there is currently no indication of outgassing which indicates that CO_2_ uptake will increase in response the continued acceleration of meltwater release from the Greenland Ice Sheet possibly facilitated by decreasing sea ice cover. The low levels of pCO_2_ contribute to the unique environmental conditions in meltwater-influenced fjords as low pCO_2_ levels can be a limiting factor for phytoplankton growth (57) and favor small-sized plankton (58) as also observed in this study.

### Long-Term Changes, Ecosystem Impacts, and Large-Scale Perspectives.

Here, we demonstrate how meltwater from several rivers connected to the Greenland Ice Sheet creates a fjord-scale gradient in physical and biogeochemical conditions, which result in distinct changes in the structure and function of the pelagic ecosystem (Fig. 7*C*). High levels of POC and turbidity in the inner fjord are the main factors driving the transition to a net heterotrophic system and provide evidence of the direct role that carbon transported by glacial rivers has on marine coastal ecosystems (3, 17). The impact on fjord ecosystems from marine-terminating glaciers has received considerable focus in recent years related to the increased productivity sustained by upwelling of subglacial discharge. However, as glaciers continue to retreat, the occurrence of land-terminating glaciers will increase and a larger proportion of the meltwater will be delivered through rivers, creating environmental conditions similar to those observed in the inner parts of Young Sound. We show that meltwater input to this region has more than doubled and the interannual variability has increased (Fig. 7*B*). Meltwater input to South and West Greenland fjords has likely increased even more (59), which suggests major but largely undocumented ecosystem change across Greenland’s fjords. This has major implications for the structure and function of the pelagic ecosystem, as we document here, with consequences for key ecosystem services such as productivity and CO_2_ uptake capacity. The high particle load during summer may also limit the distribution of benthic communities from establishing as seen from the loss of benthic biomass and diversity in glacial fjords (60, 61) and the absence of kelp forests (62). Consequently, in addition to the distinct environment created in summer during peak discharge exemplified by the current study, we hypothesize that increased discharge of meltwater with its associated particle load could reduce taxonomic and functional diversity of macrobenthic flora and fauna in Greenland fjords. These impacts could be applicable to other glaciated areas outside Greenland, and studies in both Alaska and Antarctica have documented the importance of glacial meltwater for production and structure of coastal ecosystems. However, the complexity of identifying ecosystem impacts of climate change is highlighted when runoff is considered in context with changes in sea ice cover. For example, less sea ice and more runoff may both facilitate the uptake of atmospheric CO_2_ by increasing the duration of open water and lowering the partial pCO_2_ in the surface water (Fig. 7*A*). In contrast, runoff can limit both light availability and vertical mixing, thus impairing primary production, whereas the reduction in sea ice cover can increase light availability and facilitate wind-driven mixing and thus nutrient input. In outer Young Sound, the ratio of terrigenous/riverine carbon to marine production has been estimated to be 900 /1119 t C (45). It is clear that this ratio is skewed more toward terrigenous/riverine carbon in the inner fjord and that this input has likely increased proportional to the long-term increase in total runoff. A model study has previously indicated that increased runoff to the fjord would not change the thickness of the freshwater lens (63), which implies that the area with turbid low saline surface water increases significantly as well as the region where riverine/glacial carbon is dominant.

The current study was conducted in a fjord connected to a glaciated catchment and in a region with extensive seasonal sea ice coverage. However, increase in river discharge (64) and reduction in sea ice cover (65) are circum-Arctic phenomena. The changes in Arctic catchments including permafrost thaw, tundra greening, and glacial melt combined with changes of the timing and magnitude of freshwater discharge to the ocean all have implications for the coastal marine ecosystem. And so does the reduction in ice cover that increase the potential flux of heat, wind energy, light, and greenhouse gasses between the atmosphere and coastal ocean. Since the large-scale changes in freshwater discharge and sea ice reduction are well quantified, it is now time for a major push to untangle the complex implications for coastal ecosystems. It is, for example, essential to quantify the vertical mixing and nutrient replenishment to the photic zone in different local settings (tidal amplitude, sill depth, glacier type, sea ice coverage, and runoff volume) in order to understand future changes in productivity and CO_2_ uptake. Since it is important to embed seasonal dynamics within long-term changes, such studies will clearly benefit from a multidisciplinary approach that integrates in situ processes studies, biogeochemical models, and remote sensing. Remote sensing has been essential to demonstrate the general greening of the Arctic tundra (66), and the coverage, resolution, and general accessibility of data from satellite-based sensors do now allow for a circum-Arctic analysis of the potential darkening of the coastal ocean (67) and its implications for coastal carbon cycling (68).

## Data Availability

All study data are included in article and raw data can be provided by request to the corresponding author. The data used for this publication is available on Zenodo (10.5281/zenodo.7371691).
